# Interactions between neutrophils and T-helper 17 cells

**DOI:** 10.3389/fimmu.2023.1279837

**Published:** 2023-10-18

**Authors:** Xinzou Fan, Panyin Shu, Ying Wang, Ning Ji, Dunfang Zhang

**Affiliations:** ^1^ Department of Biotherapy, State Key Laboratory of Biotherapy and Cancer Center, Collaborative Innovation Center of Biotherapy, West China Hospital, Sichuan University, Chengdu, Sichuan, China; ^2^ State Key Laboratory of Oral Diseases, National Clinical Research Center for Oral Diseases, Research Unit of Oral Carcinogenesis and Management, Chinese Academy of Medical Sciences, West China Hospital of Stomatology, Sichuan University, Chengdu, Sichuan, China

**Keywords:** neutrophils, T-helper 17 cells, autoimmune diseases, interleukin-17, neutrophil extracellular traps (NETs)

## Abstract

Neutrophils comprise the majority of immune cells in human peripheral circulation, have potent antimicrobial activities, and are clinically significant in their abundance, heterogeneity, and subcellular localization. In the past few years, the role of neutrophils as components of the innate immune response has been studied in numerous ways, and these cells are crucial in fighting infections, autoimmune diseases, and cancer. T-helper 17 (Th17) cells that produce interleukin 17 (IL-17) are critical in fighting infections and maintaining mucosal immune homeostasis, whereas they mediate several autoimmune diseases. Neutrophils affect adaptive immune responses by interacting with adaptive immune cells. In this review, we describe the physiological roles of both Th17 cells and neutrophils and their interactions and briefly describe the pathological processes in which these two cell types participate. We provide a summary of relevant drugs targeting IL-17A and their clinical trials. Here, we highlight the interactions between Th17 cells and neutrophils in diverse pathophysiological situations.

## 1 Introduction

Over the past few years, our understanding of neutrophil function has considerably progressed. Neutrophils play a role in homeostatic control as part of innate immunity and can affect adaptive immunity in tissues through activation or inhibition. The concept of T-helper 17 (Th17) cells was introduced less than 20 years ago. Due to their immunomodulatory roles in autoimmune diseases and infection resistance, Th17 cells have been the subject of substantial research. Th17 cells are potent inducers of neutrophils, recruiting neutrophils to sites of inflammation via IL-17A and promoting inflammation. The mechanisms underlying Th17 cell-mediated induction of neutrophils have been studied earlier, whereas the impact of neutrophils on Th17 cells remains largely unknown. Recent studies have reported that neutrophils regulate Th17 cells. Therefore, we reviewed the current knowledge on the mechanisms by which neutrophils modulate the impact on Th17 cells.

## 2 Neutrophils

Neutrophils comprise the majority of myeloid leukocytes in humans, constituting 50–70% of circulating leukocytes (1). Neutrophils have a short lifespan, with a specific polymorphonuclear and defined granule content (2). Neutrophils are believed to activate and regulate innate and adaptive immunity, mainly Th17 cell-driven adaptive immune responses against infections and the subsequent activation and regulation of innate and adaptive immunity against infections (3, 4). Neutrophils interact in a complex bidirectional manner with various immune cells by expressing a wide range of cytokines and immunosuppressive and immunostimulatory molecules (5, 6). The three key ways whereby neutrophils fight pathogens are phagocytosis, degranulation, and the formation of neutrophil extracellular traps (NETs) (7). The process of neutrophil phagocytosis is receptor-mediated. The neutrophil cytoskeleton rearranges, and the plasma membrane surrounds the target organism or granules to form phagocytic vesicles. Preformed granules within neutrophils fuse rapidly with phagocytic vesicles to kill and degrade target organisms or granules (8). Neutrophil phagocytosis accompanied by degranulation. Neutrophil degranulation occurs mainly at the plasma membrane, mimicking the granule–phagosome fusion. Neutrophils perform their antimicrobial functions by releasing granule-derived soluble proteins extracellularly via degranulation to activate and deliver various antimicrobial components (9). DNA, histones, and neutrophil granule proteins form net-like structures known as NETs, produced by activated neutrophils (10). NET-binding proteins are mainly cationic bactericidal proteins, including 24 proteins such as histones, elastase, cathepsin G, myeloperoxidase (MPO), proteinase 3, neutrophil defensin, calcium-binding protein, lactoferrin, and lysozyme (11, 12). Notably, NETs can be formed in two ways, i.e., NETosis and nonlytic NETosis. In NETosis or canonical death pathway, peptidylarginine deiminase-4 converts arginine in histones to citrulline after being triggered by Ca^2+^ ions. The strong positive charge on histones diminishes when arginine changes to citrulline, which weakens their strong electrostatic binding to DNA in the nucleosome, leading to chromatin decompression and NET formation. Subsequently, the neutrophil nuclear membrane begins to disintegrate, and the nucleus loses its lobules, starts to swell, and undergoes granule proteolysis. Eventually, neutrophils excrete histones and granules and deconcentrate DNA to form NETs (13, 14), which, when released, kill pathogens directly or capture them and wait for further processing by phagocytes (14–16). The second is the nonlytic form of NETosis. After exposure to *Staphylococcus aureus*, neutrophils rapidly expel chromatin and granules to form NETs. However, this process is not accompanied by neutrophil death (7). The cytoplasm left after the neutrophils excrete chromatin and granulins can continue to perform bactericidal functions by ingesting and engulfing pathogens (17). Reactive oxygen species (ROS) are not necessary for nonlytic NETosis (18).

The antimicrobial activity of neutrophils is biotoxic. When neutrophil activation is not properly controlled, pathological damage may occur in the host. Neutrophils are associated with numerous autoimmune diseases. Phenotypic and functional neutrophil aberrations, increased levels of circulating pro-inflammatory low-density granulocytes (LDGs), increased neutrophil apoptosis, and loss of neutrophil phagocytic function are observed in patients with systemic lupus erythematosus (19). Neutrophil-forming NETs are believed to be involved in atherosclerotic thrombosis (20). They play an essential role in thrombosis by promoting the deposition of hemofibrin and the formation of hemofibrin networks (21). Activated platelets induce neutrophils to form NETs (22). Impaired directed migration and function of neutrophils during sepsis leads to harmful accumulation in the lungs and liver (23, 24). In the early stages of sepsis, platelets stimulate neutrophils to release NETs that benefit the host by wrapping and eliminating pathogens. Notably, NETs and their components can harm host tissues and endothelium during severe infections and may lead to the formation of a diffuse thrombus (25). Neutrophils play a significant role in malignancies. Neutrophils undergo ferroptosis and release immunosuppressive molecules that promote tumor cell growth in the tumor microenvironment (26). Neutrophils promote tumor cell metastasis by forming NETs and encapsulating tumors in NETs, limiting tumor killing caused by cytotoxic T cells and promoting the escape of tumor cells from the immune system (27–29).

## 3 Th17 cells

The first CD4 helper T lymphocytes described were Th1 and Th2 subpopulations. Th17 cells were first conceptualized in 2005 (30, 31). This subset of lymphocytes, which differentiate from naive CD4 T cells, is known for its capacity to release interleukin (IL)-17A at high levels. Th17 cells release cytokines such as IL-17F, IL-21, and IL-22, in addition to IL-17A (32, 33). Th17 differentiation is associated with retinoic acid-related orphan receptor (ROR)γt, interferon regulatory factor 4 (IRF4), basic leucine zipper ATF-like transcription factor (BATF), and aryl hydrocarbon receptor (AHR). Notably, RORγt was shown to be the first critical regulator of Th17 differentiation. It induces the expression of IL-17A and IL-17F, and lack of RORγt leads to a functional decline in Th17 (34). Moreover, IRF4 is located upstream of RORγt, and its deficiency results in the reduced capacity of naïve T cells to upregulate RORγt (35). Both Th17 and T regulatory cells (Tregs) express AHR, and Th17 cells do so relatively more highly, and the ablation of AHR significantly lowers the expression of the Th17 effector cytokine IL-22 (36). BATF is not a Th17 cell-specific transcriptional regulator and is essential for Th17 cell differentiation and related cytokine expression (37). The differentiation of Th17 in mice can be divided into three stages: (1) due to the effects of IL-6 and transforming growth factor-beta (TGF-β) on naïve T cells in mice, the activation of signal transducer and activator of transcription 3 (STAT3) causes IL-21 production; (2) IL-21 upregulates the expression of IL-17A, IL-23R, and STAT3-dependent expression of RORγt in an autocrine manner to maintain Th17 differentiation; and (3) IL-23 derived from antigen-presenting cells interacts with the IL-23 functional receptor, promoting Th17 maintenance, proliferation, and effector activities (38–41).

Th17 cells were terminally differentiated. However, Th17 cells can transdifferentiate into certain cytokines or inflammatory environments. The difference between Th1 and Th17 subsets is not as distinct as previously believed in experimental autoimmune encephalomyelitis (EAE). Notably, T cells with an *in vitro* bias toward Th17 cells develop a Th1-like phenotype *in vivo* and can produce interferon-gamma (IFN-γ) (42, 43). Th17 cells are abundant in the lamina propria of the intestinal mucosal barrier. Short-chain fatty acids promote Treg differentiation and attenuate EAE, whereas long-chain fatty acids stimulate Th17 and Th1 differentiation and exacerbate EAE (44). Bile acid metabolites regulate Th17/Treg homeostasis in the intestine (45). In autoimmune diseases, Th17 cells transdifferentiate into Tregs, secrete IL-10, and acquire regulatory and anti-inflammatory functions (46, 47). Hypoxia modulates the Th17 phenotype. Hypoxia-inducible factor 1α is required for Th17 differentiation, mediates glycolytic activity and forkhead box P3 degradation, and regulates Th17 and Treg homeostasis (48, 49). Th17 cells are heterogeneous in their phenotype, transcription, and metabolism. Th17 cells are classified into hypermetabolic and hypometabolic subsets based on their metabolism. Hypermetabolic Th17 has strong IFN-γ expression and supports transdifferentiation to Th1-like cells (50).

Th17 cells and their associated effector cytokines play crucial roles in the pathogenesis of many autoimmune diseases and mediate defense mechanisms directed against extracellular pathogenic infections. They are essential for maintaining the homeostasis of the mucosal immune microenvironment. Hosts lacking Th17 cells are highly susceptible to fungal infections. In a mouse model of systemic candidiasis in which the IL-17A/IL-17A receptor axis was essential for the host defense system *in vivo*, Th17 protected mice from lethal doses of the oral commensal fungus *Candida albicans* infection (51). Th17- and IL-17R-deficient mice exhibited severe oropharyngeal candidiasis, and the saliva of these mice showed low *C. albicans*-killing activity (52). In addition, Th17 cells participate in host defense against *S. aureus* and *Citrobacter rodentium* in the mucus epithelium (53). Th17 cell overexpression has a detrimental effect on the host immune response. The IL-27/IL-27R complex mediates chlamydial-protective immunity by inhibiting Th17 cell overresponse and reducing neutrophil-induced inflammation (54). Th17 cells have been associated with various autoimmune diseases. Psoriasis is an inflammatory skin disease characterized by well-defined red macules and sterile pustules. Notably, IL-17A and IL-23 are key drivers of psoriasis development. Th17 cell counts are elevated and positively linked to disease activity in the blood and skin lesions (55). Patients with psoriatic arthritis often have resident Th17 cells within the synovium that overexpress IL-17A and IL-22, exacerbating inflammation and bone reconstruction. Research using mouse models of colitis has demonstrated that inflammatory bowel disease is significantly influenced by IL-17A derived from Th17 cells. Aberrant Th17 proliferative diseases produce enormous levels of pro-inflammatory cytokines, which attract additional inflammatory cells and trigger abnormal immune responses (56). In both EAE and multiple sclerosis (MS), Th17 cells play a key pathogenic role in central nervous system (CNS) inflammation in both EAE and MS. Th17 cells are abundant in the brain and spinal cord of EAE mice, as well as in the CNS lesions, cerebrospinal fluid, and peripheral blood of patients with MS, and are positively correlated with disease activity (57–60). Mice lacking Th17 cells are resistant to EAE (61). Both asthma and chronic obstructive pulmonary disease, characterized by high mucus production, share a similar immunological pathogenesis. Notably, IL-17A regulates respiratory mucus production, and high levels of IL-17A may be linked to severe intestinal and respiratory inflammation (62, 63). Th17 cells protect the body from metabolic syndrome by regulating intestinal microbiota and fat absorption (64).

The contribution of IL-17A and Th17 cells to various diseases is the basis for their clinical targeting. **Table 1** summarizes the current status of IL-17A-targeted drugs.

**Table 1:** Clinical trials using IL-17A as a target.

Drug	Disease	Phase	NCT code
Secukinumab (Cosentyx/AIN457)	Psoriasis	Completed	NCT05320159
	Psoriatic arthritis	Completed	NCT02854163
	Pityriasis rubra pilaris	Completed	NCT03342573
	Hidradenitis suppurativa	Completed	NCT03099980
	Non-infectious uveitis	Completed	NCT00685399
	Ankylosing spondyloarthritis	Completed	NCT02896127
	Rheumatoid arthritis	Completed	NCT01426789
	Dry eye	Completed	NCT01250171
	Bullous pemphigoid, pemphigoid	Completed	NCT03099538
	Multiple sclerosis	Terminated	NCT01874340
	Asthma	Terminated	NCT01478360
	Necrobiosis lipoidica diabeticorum	Terminated	NCT03791060
	Type 1 diabetes mellitus	Terminated	NCT02044848
	Lupus nephritis	Phase III	NCT04181762
Ixekizumab (LY2439821)	Psoriasis	Completed	NCT03073213
	Rheumatoid arthritis	Completed	NCT00966875
	Hidradenitis suppurativa, Acne inversa	Completed	NCT04979520
	Pityriasis rubra pilaris	Completed	NCT03485976
	Type1 diabetes mellitus	Phase II	NCT04589325
	Spondylitis, ankylosing	Phase III	NCT01870284
	Major depressive disorder	Phase II	NCT04979910
Brodalumab	Psoriasis	Completed	NCT04149587
	Ankylosing spondylitis	Completed	NCT03355573
	Psoriasis vulgaris, Psoriatic arthritis, Psoriatic erythroderma	Completed	NCT04183881
	Psoriatic arthritis	Completed	NCT02024646
	Systemic sclerosis	Phase I	NCT04368403
	Crohn’s disease	Terminated	NCT01150890
Bimekizumab	Hidradenitis suppurativa	Completed	NCT04242498
	Psoriatic arthritis	Completed	NCT03895203
	Chronic plaque psoriasis	Completed	NCT03025542

This table summarizes information on a selection of IL-17A-targeted drugs used in clinical trials at this stage and provides an overview of the indications for these drugs and the stage of clinical trials they are in. https://clinicaltrials.gov.

Secukinumab is a high-affinity, fully humanized monoclonal immunoglobulin (Ig)G1κ antibody that selectively binds to IL-17A and blocks IL-17A from binding to its receptor, thereby controlling the progression of IL-17A-associated diseases, such as psoriasis, psoriatic arthritis, ankylosing spondylitis (65–68). Secukinumab has a blocking effect and was clinically effective in a phase III clinical trial of plaque psoriasis. Psoriasis area and severity index (greater than or equal to 75) scores (PASI75) decreased by at least 75% compared to placebo and Etanercept (69). Histologically, epidermal hyperplasia reduced rapidly, and psoriasis-associated transcriptome expression was significantly reduced in genomic analysis (70). Secukinumab has been shown to be effective in controlling psoriatic arthritis. In phase III clinical trials, subcutaneous injections of 150 or 300 mg secukinumab effectively treated psoriatic arthritis. In the FUTURE trial, both 150 and 300 mg doses of secukinumab significantly improved the symptoms of psoriatic arthritis, with higher American College of Rheumatology 20% response criteria (ACR20) at 16 and 24 weeks of treatment than in patients using placebo (71–75). The proportions of patients achieving greater than or equal to 50% improvement in ACR response criteria (ACR50) and patients achieving improvements in PASI75 and PASI90 were significantly higher than in patients using placebo. Notably, ACR20 response rates at 5 years can still be as high as 70–74% (76, 77). Secukinumab improved the clinical signs and symptoms compared with those obtained with placebo use. Moreover, the improvement was sustained over a longer period than with the use of tumor necrosis factor (TNF) antagonists, making secukinumab an effective treatment modality that can be used as an alternative to TNF antagonists.

Ixekizumab is a high-affinity, recombinant humanized monoclonal IgG4κ antibody indicated for the treatment of psoriatic arthritis and moderate-to-severe plaque psoriasis by binding specifically to IL-17A and blocking IL-17A activity, reducing inflammation and achieving relief in psoriasis symptoms (78). Ixekizumab was significantly more effective than placebo in phase I and II clinical trials of psoriasis treatment. In a phase I trial, 150 mg of ixekizumab every 2 weeks resulted in maximum disease control within 6 weeks (79). Phase II trials reduced the concentration to 75 mg every 2 weeks and achieved satisfactory disease control (80). Ixekizumab is indicated to treat genital psoriasis, generalized pustular psoriasis, and psoriatic arthritis. For treating patients with genital psoriasis, 80 mg of ixekizumab was administered every 2 weeks for 12 weeks. At 12 weeks of treatment, genital pruritus scores decreased in 60% of patients using ixekizumab compared with that in patients using placebo, and 73% of patients using ixekizumab had genital pruritus scores of “0” or “1” (81). The clinical phenotype and genetic background of generalized pustular psoriasis differ from those of normal psoriasis and are uncommon in the clinic; therefore, there are not many clinical subjects of different biological agents in generalized pustular psoriasis. Promising therapeutic efficacy with limited adverse effects was observed in a German patient treated with ixekizumab for one year (82). In a psoriatic arthritis clinical trial, patients were randomized into the following four groups: 80 mg ixekizumab every 2 weeks group, 80 mg ixekizumab every 4 weeks group, 40 mg adalimumab every 2 weeks group, and a placebo group. The trial was concluded at 24 weeks, and the ACR20 rates were 62.1, 57.9, 57.4, and 30.2% for the corresponding groups, respectively (83). Ixekizumab is effective in the treatment of psoriatic arthritis.

Brodalumab is a humanized IL-17RA IgG2 monoclonal antibody. Brodalumab inhibits IL-17A downstream signaling by binding to a shared subunit of the IL-17 receptor complex and blocking multiple IL-17 family cytokines (84). Brodalumab is indicated for the treatment of moderate-to-severe plaque psoriasis. In the AMAGINE-1 phase III clinical trial, patients with plaque psoriasis were divided into the following three groups: 140 mg brodalumab every 2 weeks, 210 mg brodalumab every 2 weeks, and placebo. The PASI75 and static Physician’s Global Assessment of 0 or 1 (sPGA 0/1) ratios of these three groups were determined at 12 weeks. The PASI75 and sPGA 0/1 ratios increased significantly in subjects using Brodalumab (85). Based on the outcomes of the AMAGINE-2,3 phase III clinical trials, brodalumab (210 mg every 2 weeks) had a higher rate of PASI100 than that with ustekinumab use at 12 weeks. In addition, brodalumab provided long-term, high-level skin clearance in patients with moderate-to-severe plaque psoriasis. At 52 weeks, patients administered 210 mg brodalumab every 2 weeks rapidly achieved high levels of complete and sustained skin clearance (86). Brodalumab is effective in treating psoriatic arthritis. At 12 weeks, patients treated with 140 and 280 mg brodalumab had a higher ACR20 rate than those using placebo (87).

Bimekizumab was the first IL-17A/IL-17F dual-target inhibitor for moderate-to-severe plaque psoriasis. In the BE VIVID phase III clinical trial, patients with moderate-to-severe plaque psoriasis were divided into three groups: a 320 mg bimekizumab every 4 weeks group, a 45 or 90 mg (chosen based on body weight) ustekinumab group at weeks 0 and 4 and every 12 weeks subsequently, and a placebo group. At 16 weeks, 85% of the patients in the bimekizumab group achieved PASI90, a much higher proportion than those in the ustekinumab and placebo groups. Bimekizumab has performed well in treating moderate-to-severe plaque psoriasis, with a therapeutic efficacy superior to that obtained with ustekinumab use (88). In the BE OPTIMAL phase III trial, patients with psoriatic arthritis were divided into the following three groups: 160 mg bimekizumab every 4 weeks group, placebo group, and 40 mg adalimumab every 2 weeks group. At week 16, subjects in the placebo group were administered 160 mg of bimekizumab every 4 weeks. The proportion of patients with ACR50 was counted at week 16, and a significantly higher number of bimekizumab-treated patients than those in the placebo group achieved ACR50. Improvements in joint, skin, and imaging outcomes were relatively more pronounced in the bimekizumab-treated patients than in the placebo group (89).

## 4 Effects of Th17 cells on neutrophils

**Figure 1:**
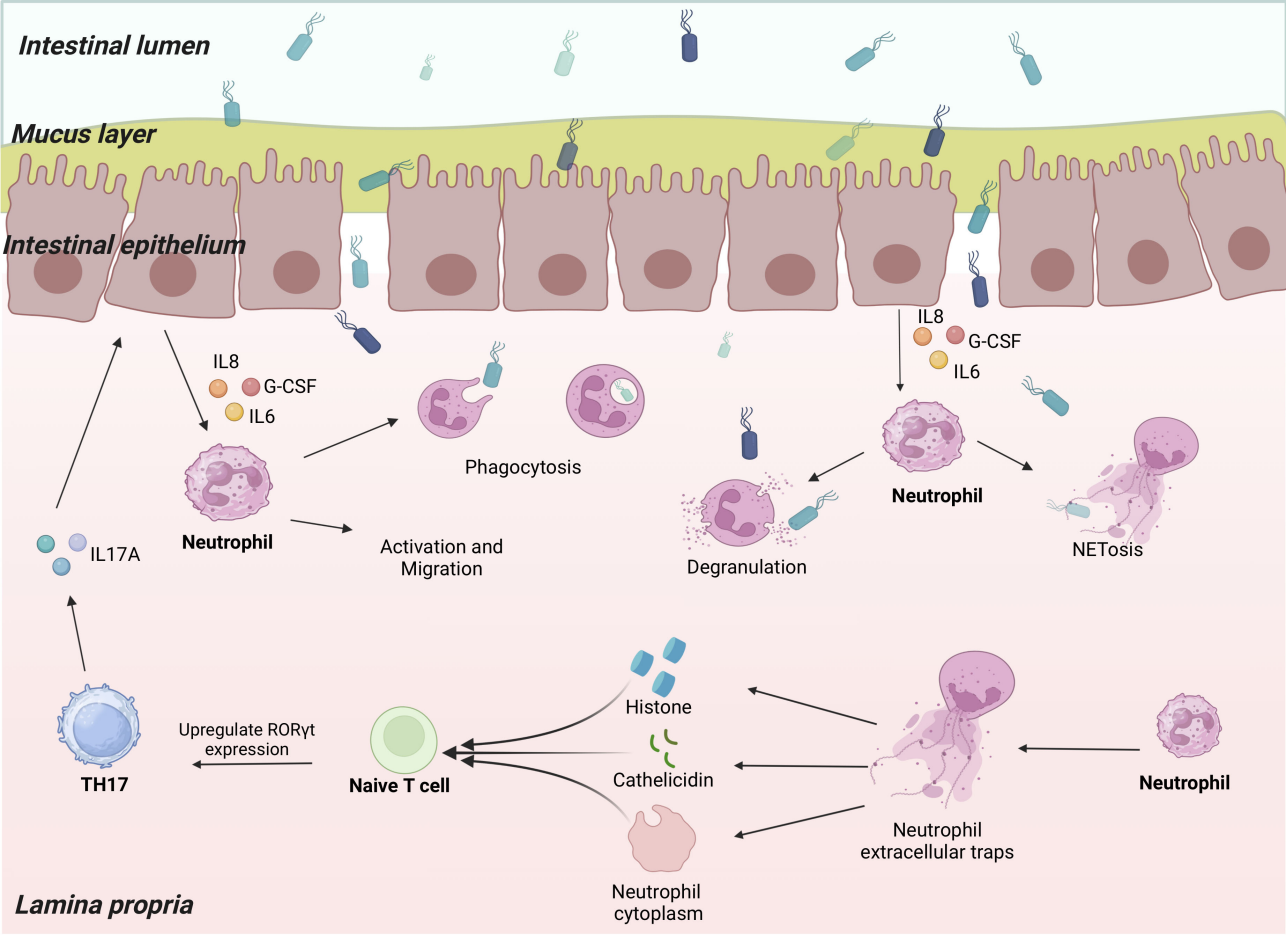
Neutrophils communicate with Th17 cells. Th17 cells secrete IL-17A to act on epithelial cells when the organism is infected. The epithelial cells produce a series of neutrophil chemokines that induce neutrophil activation and migration to the site of infection. Neutrophils carry out anti-infective functions through phagocytosis, degranulation, and NETosis. Through their histones, cathelicidin, and the cytoplasm remaining after NETosis, Neutrophil extracellular traps (NETs) promote naïve T cell upregulation of RORγt expression, thereby promoting Th17 differentiation.

Th17 cells affect neutrophils via IL-17A production. Increasing the levels of granulocyte colony-stimulating factor (G-CSF), IL-17A encourages the synthesis of granulocytes and the mobilization of mature neutrophils from the bone marrow. Through peripheral blood circulation, neutrophils travel to the area of infection or inflammation and perform their effector functions (**Figure 1**).

IL-17A does not act directly on neutrophils; however, it recruits them by acting on a variety of nonimmune cells (e.g., epithelial cells, endothelial cells, and fibroblasts), causing them to release neutrophil chemokines, such as chemokine (C-X-C motif) ligand (CXCL) 1, CXCL6, and CXCL8 (90). Neutrophils in blood vessels sense these chemokines via the C-X-C motif chemokine receptor (CXCR) 1, which activates signaling downstream of vasodilator-stimulated phosphoprotein, phosphoinositide 3-kinase, and Src family kinase, mediating directional migration of neutrophils (91) (92). In addition, activated neutrophils produce matrix metalloproteinases that cleave CXCL8 family chemokines and enhance their chemotaxis (92). Overexpression of IL-17A upregulates neutrophil-targeted chemokine expression, which, in turn, increases neutrophil infiltration. Conversely, knockdown of IL-17A expression or defective IL-17A receptors results in downregulated chemokine expression and inhibition of neutrophil infiltration (91). However, IL-17A acts directly on neutrophil CXCR2 to induce neutrophil chemotaxis (93).

Moreover, IL-17A exerts a granulogenic effect (91). It intervenes in neutrophil generation by upregulating G-CSF expression, which plays a crucial role in neutrophil development. For example, G-CSF directs the differentiation of committed progenitors to myeloid lineage, promotes the proliferation of neutrophil precursors, reduces the time required for neutrophils to pass through the compartment, and promotes the release of mature neutrophils from bone marrow (94, 95). During inflammation, IL-17A, derived from Th17 cells, promotes G-CSF production, which acts on neutrophils in response to inflammation. After the inflammation subsides, neutrophils undergo apoptosis, and these apoptotic neutrophils are cleared by nearby phagocytes (such as macrophages) (94). This process is accompanied by a decrease in IL-23 levels, which, in turn, leads to a reduction in Th17-derived IL-17A production, resulting in decreased G-CSF synthesis (96, 97). Defects in G-CSF, induced by anti-IL-17A antibodies, result in elevated levels of the chemokine stromal cell-derived factor (SDF)-1α, a retention factor for neutrophils to reside in the bone marrow. Elevated levels of SDF-1α interfere with bone marrow mobilization of neutrophils, leading to a decrease in the number of circulating neutrophils (98).

The following subsections describes how Th17 cells affect neutrophils in various immune microenvironments.

### 4.1 Effects of Th17 cells on neutrophils in psoriasis

Characterized by epidermal proliferation and inflammation, psoriasis is a chronic relapsing inflammatory skin disease (55). The etiology of psoriasis is greatly influenced by the IL-23/IL-17A axis and, to a certain extent, reflects disease severity (99). Moreover, IL-23 activates IL-17A-producing Th17 cells and enhances their cytokine production capacity and pathogenicity. In patients with psoriatic arthritis, high levels of IL-23 and IL-17A are present in the synovium, and tissue-retained Th17 cells highly express IL-22 and IL-17A. The production of granulocyte-macrophage CSF (GM-CSF) and several chemokines, including CXCL8, is promoted in response to the IL-23/IL-17A axis (99). CXCL8 promotes neutrophil recruitment and migration, whereas GM-CSF and G-CSF replenish circulating neutrophils, which are exhausted during infiltration into the epithelium (100). In patients with psoriasis, skin lesions, serum Th17 cell counts, and IL-17A levels were positively correlated with disease activity (101). Various immune and nonimmune cells are involved in the pathogenesis of psoriasis. In addition to driving epidermal hyperproliferation and activating keratin-forming cells, IL-17A promotes the production of several cytokines and chemokines, such as the chemokine CXCL8. CXCL 8 induces neutrophil migration to lesion sites and promotes inflammation (102). Notably, IL-17A induces epithelial cells to express IL-36, contributing to neutrophil inflammatory activity and migration (103, 104). In addition, IL-17A has an apoptosis-inhibiting effect on neutrophils and promotes the production of IL-1, IL-6, and TNFα. Notably, TNFα activates neutrophils and promotes neutrophil infiltration into the epithelium, amplifies and propagates inflammation, promotes characteristic changes in psoriasis symptoms, and creates a self-sustaining inflammatory cycle (100). Neutrophils are a source of IL-17A at psoriatic lesions (99, 105). Neutrophil-derived IL-17A is a potential drug target for the treatment of psoriasis. Targeted drugs prevent neutrophils from interacting with keratin-forming cells and entering the lesion site (101). In lesions of progressive psoriasis, large aggregates of neutrophils are often observed at the epidermal site, forming neutrophilic pustules such as Kogoj spongiform pustules and Munro’s microabscess, among other typical pathologic changes (100). Neutrophils infiltrating the epidermis produce NETs that act directly on keratinocytes and are involved in the maintenance and amplification of the inflammatory environment in the skin. In addition, MPO and NETs released during neutrophil degranulation are involved in ROS generation, protein hydrolysis of inflammatory mediators, and self-antigen formation during psoriasis (106).

### 4.2 Effects of Th17 cells on neutrophils in asthma

Asthma is a common chronic noncommunicable respiratory disease characterized by chronic airway inflammation, hyperresponsiveness, variable reversible airflow limitation, and airway remodeling due to the prolonged course of the disease. Approximately 334 million people are affected by asthma (107). The traditional view is that dysregulation of Th2 immunity is the primary cause of asthma. However, recent studies have revealed that Th17 cell-secreted IL-17A plays a role in the development and progression of neutrophilic asthma. Patients with severe asthma have higher levels of IL-17A in their airways when their diseases are more severe (108). Th17 cells secrete cytokines, such as IL-17A, and recruit neutrophils by acting on airway epithelial cells expressing the neutrophil chemokines CXCL1 and CXCL8. Neutrophils that receive recruitment signals migrate to the airways and produce cytokines, such as IL-6, CXCL8, G-CSF, and macrophage inflammatory protein-2, which induce neutrophilic inflammation in the airway (103, 109). Most patients with asthma control their symptoms using glucocorticoid therapy. However, in patients with steroid-resistant asthma (SRA), glucocorticoid use does not adequately control the symptoms (110). Patients with SRA have large neutrophil infiltrates in the airways and high Th17/IL-17A expression (111, 112). Neutrophilic asthma exhibits significant steroid resistance and IL-17A dependence (113). Glucocorticoid receptor-β (GR-β) overexpression is linked to steroid resistance in patients with SRA. The development of steroid resistance may be facilitated by GR-β, which does not bind to corticosteroids. Notably, IL-17A induces GR-β expression in the airway epithelium of patients with asthma and generates steroid resistance in peripheral blood mononuclear cells, resulting in SRA (114). Mice overexpressing RORγt exhibit steroid-insensitive neutrophilic inflammation. Anti-IL-17A antibody, CXCR2 agonist, or anti-IL-6R antibody effectively suppress airway hyperresponsiveness and neutrophilic inflammation in RORγt-overexpressing mice (115). Airway remodeling is a crucial pathological hallmark of asthma. IL-17A, derived from Th17 cells, can activate and secrete neutrophil chemokines, such as IL-6 and CXCL8, by promoting the activation of various cells, such as epithelial cells, fibroblasts, and neutrophils, which causes neutrophils to release a variety of bioactive substances, such as neutrophil elastase, in the airways to mediate lung tissue destruction and mucus hypersecretion (112).

### 4.3 Effects of Th17 cells on neutrophils in other diseases

Inflammatory resorption of the bone tissue supporting the teeth is a hallmark of the chronic, progressive, and destructive disease periodontitis. According to previous studies, bacteria are the primary cause of periodontitis. Th17 cells are crucial for the progression of periodontitis and correlate with disease severity (116). Th17-derived IL-17A is involved in bone destruction in periodontitis. In the presence of IL-17A, fibroblasts and epithelial and endothelial cells of gingival tissue produce numerous inflammatory mediators, e.g., IL-6, CXCL8, IL-1β, granulocyte chemotactic protein, and GM-CSF, promoting neutrophil-mediated inflammation and causing tissue damage and alveolar bone resorption (117–121). In an experimental periodontitis model, i.e., IL-17A receptor-deficient mice infected with *Pseudomonas gingivalis*, neutrophil recruitment decreased at the site of periodontal lesions; however, bone loss was increased (122).

Th17 cells are present in the tumor microenvironment, and excessive inflammation caused by Th17 cells may be associated with tumorigenesis (90). Notably, IL-17A activates and recruits antitumor immune cells, such as neutrophils, macrophages, natural killer cells, and CD8^+^ T cells, to exert antitumor effects (123). In breast cancer, Th17 cells promote tumor development by recruiting neutrophils via IL-17A (124).

In necrotic glomerulonephritis, IL-17A deficiency leads to impaired neutrophil recruitment to the glomerulus, which, in turn, downregulates MPO and improves glomerulonephritis (125).

The Th17 cell counts are higher in the decidua than in the peripheral blood and correlate positively with neutrophil counts. Th17 cells induce protective immunity against extracellular microbes through neutrophils (126, 127).

## 5 Effects of neutrophils on Th17 cells

Numerous studies have documented mechanisms underlying Th17 cell-mediated induction of neutrophil chemotaxis. In recent years, as neutrophils have been studied relatively more intensively, researchers have found that they interact with Th17 cells.

The formation of NETs is a major mechanism underlying neutrophil activation to fight pathogens. DNA and histones constitute most NETs. Histones are either free or bound to DNA. Free histones are relatively more pathogenic and have pro-inflammatory effects (128–130). Large numbers of NETs and histones are present in patients with autoimmune diseases. However, the exact function of these components remains unknown (131). Wilson et al. (132) co-cultured NETs with naïve T cells under Th17-differentiation conditions and found that NETs promoted Th17 differentiation in a concentration-dependent manner. This effect is eliminated upon the application of histone inhibitors. Upregulation of RORγt expression leads to the promotion of Th17 differentiation by NET-derived histones. Specifically, histones activate toll-like receptor 2 in naïve T cells, which phosphorylates STAT3, thereby facilitating Th17 differentiation. The neutrophil antimicrobial peptide cathelicidin has potent antimicrobial activity and immunomodulatory effects and can be released from neutrophils upon degranulation, NET formation, and necrotizing death. Minns et al. (133) reported that cathelicidin derived from neutrophils released by secondary lymphoid organs in mice promotes Th17 cell differentiation in a TGF-β-dependent way by upregulating the expression of AHR and RORγt. Cathelicidin dramatically reduced Tbet expression while promoting STAT3 and Smad2/3 phosphorylation in the presence of TGF-β. Neutrophils and Th17 cells converge at sites of inflammation by producing chemokine C-C motif ligands 20/22, IL-17A, and CXCL8 (134). During severe asthma, neutrophils form nonlytic NETosis, and the remaining cytoplasm can indirectly induce Th17 cell differentiation in the lung (135). Toll-like receptor 8-dependent activated neutrophils can produce the Th17-inducing differentiation factor, IL-23, and their culture supernatant induces naïve T cells to differentiate into Th17 cells (136). *In vitro*, Th17 differentiation is promoted by neutrophil-derived elastase, which converts dendritic cell-derived CXCL8 into the Th17-promoted form (137). Neutrophils from patients with autoimmune diseases are defined as LDGs (19). This class of neutrophils has pro-inflammatory properties that promote the differentiation and proliferation of Th17 cells (2, 138–140). They are stimulated by IFNs and promote Th17 differentiation by upregulating the levels of costimulatory molecules and major histocompatibility complex II (141). In addition, IL-17A recruits neutrophils to lymph nodes during the Th17 response, providing additional neutrophil-derived IL-1β for Th17 differentiation (91) (**Figure 1**).

In addition to Th17 cells, neutrophils regulate other T cells. Neutrophils are susceptible to ferroptosis. Ferroptosis-induced oxidation of phosphatidylethanolamine and lecithin inhibits T cell proliferation (26). In sepsis, the production of macrophage-1 antigens by neutrophils inhibits T cell proliferation (142). Neutrophils can disrupt T cell function and differentiation through ROS, programmed cell death-ligand 1, and arginase overexpression to degrade arginine, which is necessary for the activation and proliferation of T cells (143–147). By expressing the suppressor surface protein CD10, neutrophils can interfere with T cell function (148). During lung infection, neutrophils inhibit T cell recruitment and activation and promote the sustained expansion of microbes in the lung (149, 150). During influenza, neutrophils promote T cell function and migration to the sites of infection (151, 152). *In vitro*-cultured T cells can recognize NET components through T cell receptors, lowering the threshold for T cell activation (153).

## 6 Conclusions

Neutrophils and Th17 cells interact closely under physiological conditions. A closed loop is formed between the two via the IL-23/IL-17A axis to maintain homeostasis in the immune microenvironment. The disruption of this regulatory loop can lead to a local imbalance in immune homeostasis, resulting in inflammatory or severe pathological reactions. Extensive research has been conducted on the effects of Th17 cells on neutrophils. However, research on how neutrophils intervene with Th17 cells in a complex immune microenvironment is still in the early stages of development. Herein, we summarize how neutrophils affect and regulate Th17 cells. The study of the relationship between neutrophils and Th17 cells has considerable potential for clinical applications because of the complex relationship between the two cells and their synergy in the immune microenvironment. Future immunotherapy may depend on targeting the different biological roles of neutrophils and their effects on T cells to develop new treatments for tumors and chronic inflammatory diseases. Th17 cells are plastic, and their role in tumors is highly dependent on the tumor microenvironment. They are recruited to the sites of malignant tumors. Exploiting the interaction of neutrophils with Th17 cells, inducing the transformation of Th17 cells in specific environments, and enhancing the Th17 antitumor response may be effective strategies for the future development of cancer immunotherapies. Neutropenia, an adverse event, has been reported in a few clinical trials of drugs targeting IL-17A, summarized in this review. Neutropenia may increase the risk of infections in patients. Reducing the incidence of neutropenia as an adverse event while targeting IL-17A and reducing the risk of infections in patients will be the focus of future studies.

## Author contributions

XF: Writing – original draft. PS: Writing – original draft. YW: Writing – review & editing. NJ: Writing – review & editing. DZ: Writing – review & editing, Conceptualization, Funding acquisition.
